# Photosynthetic Mechanisms of Metaxenia Responsible for Enlargement of *Carya cathayensis* Fruits at Late Growth Stages

**DOI:** 10.3389/fpls.2020.00084

**Published:** 2020-02-19

**Authors:** Ren Huang, Yun Zhang, Qixiang Zhang, Jianqin Huang, Heikki Hänninen, Youjun Huang, Yuanyuan Hu

**Affiliations:** State Key Laboratory of Subtropical Silviculture, Zhejiang A & F University, Hangzhou, China

**Keywords:** Heterogeneous pollination, metaxenia, fruit photosynthesis, fruit quality, carbon requirement

## Abstract

Fruits of hickory (*Carya cathayensis*) are larger and their peel is greener after interspecific pollination by pecan (*Carya illinoinensis*; later pp fruits) than after intraspecific pollination by hickory (later ph fruits). Previous studies have found little genetic differences between offspring and their maternal parent, indicating that the observed trait differences between pp and ph fruits are due to metaxenia. Fruit development depends on the amount of photosynthetic assimilate available. Since there is no difference in photosynthesis of the associated leaves between pp and ph fruits, the larger size of the pp fruits might be attributed to changes in fruit photosynthesis caused by the different pollen sources. To elucidate to the photosynthetic mechanisms behind the metaxenia effect on fruit development in hickory, the effects of intraspecific and interspecific pollination regimes were examined in the present study. We observed the photosynthetic capacity in the peel of fruits and the related ecophysiological and morphological traits of both ph and pp fruits over a period of 120 days after pollination. Significant differences in the appearance and dry weight between ph and pp fruits were observed at 50 days after pollination (DAP). More than 70% of dry matter accumulation of the fruits was completed during 60–120 DAP, while the true photosynthetic rate of the associated leaves significantly decreased by about 50% during the same period. In several cell layers of the peel, the number of chloroplasts per cell was significantly higher in pp than in ph fruits. Similarly, the ribulose 1, 5-bisphosphate carboxylase (Rubisco) activity, the total chlorophyll content, and the nitrogen content were all significantly higher in pp than in ph fruits during all growth stages; and all of these physiological quantities were positively correlated with the gross photosynthetic rate of the fruits. We conclude that the enhanced photosynthetic capacity of pp fruits contributes to their fast dry matter accumulation and oil formation. This result will provide a theoretical basis for improving hickory fruit yields in practical cultivation.

## Introduction

Both *Carya cathayensis* (hickory) and *Carya illinoinensis* K. Koch (pecan) in the family Juglandaceae are important and widely planted nut tree species in Zhejiang and Anhui provinces in China. Due to its seeds' high nutritional value and unique flavor, hickory has become increasingly popular in southern China in recent years. Pecan, which originates from North America, has been cultivated in large areas of China due to its wide adaptability and suitability. Different fruiting characteristics of hickory and pecan, including differences in maturation time, size, and kernel components, allow growers the opportunity to cross these two species, and thereby possibly achieve heterosis. Effects of interspecific pollination have been recorded in hickory since the 1960s. In particular, greener peels and larger fruit size have been observed after interspecific pollination with pecan pollen (Li et al., 1982). Previous studies using molecular tools (Wang et al., 2010) and cytological investigations (Zhang et al., 2012) have shown that the effect of interspecific pollination is not caused by genetic changes, indicating that it is the result of metaxenia. The concept of metaxenia denotes all those cases when pollen type has effects on the fruit tissues of maternal origin, such as the pericarp and other fruit components, which by definition do not show any genetic effects of the pollen parent (Swingle, 1928; Denney, 1992). However, the metaxenia mechanism responsible for enlarged fruit size in hickory is still unknown.

It has been suggested that metaxenia allows fruits to reach their full yield potential (Qi et al., 2007; Miller et al., 2011). Examples of this include pecan nut, pistachio nut, chestnut and avocados (Cran and Iwakiri, 1980; Marquard, 1988; Sedgley and Griffin, 1989; Garner and Lovatt, 2016). Numerous research reports have documented the role of the pollen source on fruit set and yield as well as on several fruit characteristics, such as the time required for fruit development, the fruit appearance, and fruit components (Mizrahi et al., 2004; Mohammadi et al., 2017; Jahromi et al., 2019). However, very few studies have been carried out on the mechanisms of metaxenia. In earlier studies, pollen parent effects on hormone contents (Nie and Liu, 2002; Seal et al., 2013), or enzyme activities (Chen et al., 1999); and male chemical signals (Piotto et al., 2013) have been suggested to be included among the mechanisms of observed cases of metaxenia.

Fruits are dependent on assimilates supplied by the photosynthetic organs during their development (Cocaliadis et al., 2014). Furthermore, the fruits and the surrounding pericarp/seed coat have developed mechanisms to refix some of the respired carbon (Quebedeaux and Chollet, 1975), thereby providing an important additional contribution to meet the carbon requirements of the seeds and fruits (Whiley et al., 1992; Hu et al., 2012; Hua et al., 2012; Hu et al., 2018). Similarly to the chloroplasts in leaves, those in fruits contain proteins involved in light-harvesting complexes, electron transfer, and CO_2_ fixation (Hetherington et al., 1998; Carrara et al., 2001). Several investigators have reported that Rubisco activity, nitrogen content, and photosynthetic pigments are involved in determining the photosynthetic capacity of fruits (Xu et al., 1997; Chen and Cheng, 2007; Hu et al., 2012; Hua et al., 2012; Hu et al., 2018). Using the photosynthetis inhibitor DCMU (3-(3,4-dichlorophenyl)-1,1-dimethylurea) to prevent ear photosynthesis in wheat (*Triticum aestivum* L.), ear photosynthesis was estimated to contribute to grain filling by 22%−45% (Maydup et al., 2010). Similarly, fruit photosynthesis in both hickory and pecan significantly contribute to meeting the carbon requirements at the late growth stages (Xu et al., 2016). Furthermore, since there is no difference in the photosynthetic rates between associated leaves of the fruits pollinated with different pollen parents, the enlarged fruit size caused by interspecific pollination is likely due to difference in pericarp photosynthesis of fruits pollinated with different pollen sources. Hence, a better understanding of the traits and mechanisms of fruit photosynthesis pollinated by different pollen parents is essential for understanding the mechanism of metaxenia in hickory.

Here we studied the effects of interspecific pollination on fruit photosynthesis in hickory. To this end, fruit development was initiated by two pollination regimes, one by intraspecific hickory pollen (hickory × hickory, later ph fruits) and the other by interspecific pecan pollen (hickory × pecan, later pp fruits). The aims of the present study were 1) to elucidate the photosynthetic mechanisms behind the metaxenia effect on fruit photosynthesis during the fruit development, and 2) to evaluate the role of fruit photosynthesis in determining the differences in dry matter accumulation and quality of the fruits between the two pollination regimes. The results will improve our understanding of the mechanism underlying metaxenia.

## Materials and Methods

### Plant Materials

Ten grafted hickory (*C. cathayensis* Sarg.) trees with 2nd-year Hunan hickory as rootstock and 1st-year hickory as scion were studied at Zhejiang Agricultural and Forestry University, Lin'an, China (30°12'N, 119°20'E). The trees were grafted in 2008, and they have born fruit since 2012. The trees were grown using standard practices. All of the female flowers were bagged with sulfuric acid paper bags (7 cm × 6.5 cm × 1 cm), and the male inflorescences were removed before the start of pollen dispersal during late April in 2015 and 2016. Female inflorescences were hand-pollinated using injection needles on May 1^st^, 2015, and May 3^rd^, 2016. Pollen of hickory and pecan was collected from Linlong Mountain in Lin'an city. From each grafted maternal parent tree, two adjacent branches from the same height and with similar growth were sampled for the experiment. Flowers in the two branches were pollinated with intraspecific hickory pollen (ph fruits) and interspecific pecan pollen (pp fruits), respectively. Prior to start of the measurements, both ph and pp fruits were thinned to one fruit per cluster and labeled on June 5^th^, 2015, and July 7^th^, 2016, respectively. Measurements of gas exchange and other physiological parameters were done randomly from the outer part of the crown at a height of around 1.5−2.0 m. Four trees were used in the measurements of gas exchange and the other related physiological parameters, the other six trees were used in the photosynthesis inhibitor (DCMU,3-(3,4-dichloropheny)-1,1-dimethylurea) experiment. In all measurements one tree was considered as one replicate.

### Surface Area and Dry Biomass

The length (L), width (W), and thickness (T) of fruits were measured with a digital caliper. The surface area of fruits was determined according to Xu et al. (2016). The dry biomass (oven-dried at 60°C for 48 h to constant weight) of the fruit was determined with an electronic balance. The surface area and biomass of each fruit were measured after the gas exchange measurements on it were completed.

### Gas Exchange

Gas exchange of fruits was measured with an LI-6400 portable photosynthesis system (Li-COR, Lincoln, NE, USA). Measurements were carried out from 08:00 to 11:00 h and from 14:00 to 16:00 h. A conifer chamber (6400-22, LI-COR) with an 18-RGB light source (LI-COR, Lincoln, NE, USA) was used with fruits, and a normal 2 × 3 cm chamber with a 6400-02 (LI-COR) LED light source was used with leaves. Both the leaves and the fruits remained attached to the stem during measurements. Leaves or fruits enclosed in the chamber were first exposed to photosynthetically active radiation (PAR) of 1300 μmol m^-2^ s^-1^ for 20 min, then the net photosynthetic rate (P_net_) of the leaf or fruit was determined when CO_2_ flux in the chamber had stabilized. Immediately following measurements in the light, the dark respiration rate (R_dark_) of the leaf or the fruit was recorded as the CO_2_ exchange rate at a PAR of 0 μmol m^-2^s^-1^ for at least 30 min once the CO_2_ flux in the chamber had stabilized. The light respiration rate (R_light_) of the leaf or fruit denotes the respiration rate of the leaf or fruit under illumination. It has been reported that leaf mitochondrial respiration is reduced by 20%–130% in the light relative to that in the darkness (Heskel et al., 2013; Niinemets, 2014). All methods for estimating R_light_ involve assumptions. The simplest assumption often made is that R_light_ = R_dark_ (Kromdijk et al., 2010; Wohlfahrt and Gu, 2015; Bellasio et al., 2016). Similarly, in the present study we assumed that R_light_ equals R_dark_ in both the leaves and fruits. Therefore, the true photosynthesis (P_true_) of the leaf or fruit was calculated as P_net_ plus R_dark_ (Wohlfahrt and Gu, 2015). During the measurements, the chamber temperature was controlled at 28–30°C and CO_2_ exchange was monitored at an ambient CO_2_ concentration of about 400 μmol mol^-1^. For the leaves, we used the standard method of expressing P_net_ and R_dark_ per one-sided leaf area, so that the leaf area was taken as one-half of the total two-sided surface area of the leaf. Both the leaves and fruits were placed horizontally in the chamber during the measurements. In order to get comparable results for leaves and fruits, the gas exchange rates obtained for fruits were divided by half of the surface area of the fruit when expressing the rates per unit surface area. All of the gas exchange measurements were made on three leaves or fruits from each of four sampled trees.

### Microscopy of the Chloroplast Ultrastructure

Tissue samples (pseudo-peel) for the analysis of the chloroplast number per cell layer were taken from an individual fruit from each of the four trees at 84 DAP (July 24^th^, 2017). The samples were immediately fixed in 2.5% (v/v) glutaraldehyde (0.1 M phosphate buffer, pH 7.0) for at least 4 h once cut from the trees. The samples were then immersed in a 1% (v/v) osmium tetroxide for postfixation. The specimens were dehydrated using a graded series of ethanol and embedded in epoxy resin for ultrathin sectioning and transmission electron microscopy (H7650, HITACHI, Tokyo, Japan). The chloroplast numbers per cell were recorded from the uppermost first layer to the sixth layer of the pseudo-peel of the fruit. The sizes of the chloroplasts and cells from each layer were also determined.

### Chlorophyll Content

Discs of a given area of pseudo-peel (about 25 mm^2^) were taken from three fruits of each replicate tree. The pseudo-peel samples were ground in a ceramic mortar and transferred to a centrifuge tube with 5 ml 95% (v/v) alcohol (100%, Sinopharm Chemical Reagent Company, Shanghai, China) in the dark for 24 h at 25°C until the peel was blanched. Absorbance of the supernatant was measured with a spectrophotometer (UV-2550, Shimadzu, Kyoto, Japan) at 649, 664, and 470 nm after centrifugation. The total chlorophyll content was then calculated according to Lichtenthaler (1987).

### Nitrogen Content

The pseudo-peel samples of the three fruits from each of four sampled trees were dried and ground, and then the total nitrogen content was determined with an azotometer (Kjeltec-2300 FOSS, Sweden) according to the micro-Kjeldahl method (Schuman et al., 1972).

### Rubisco Activity

The extraction of Rubisco was carried out according to Chen and Cheng (2007). Approximately 0.2 g of pseudo-peel samples of three fruits from each of the four sampled trees were ground with a liquid nitrogen pre-cooled mortar and pestle in 1.5 ml extraction buffer [50 mM Hepes-KOH pH 7.5, 10 mM MgCl_2_, 2 mM EDTA, 10 mM dithiothreitol (DTT), 10% (v/v) Glycerol, 1% (w/v) bovine serum albumin (BSA), and 1% Triton X-100]. The extract was centrifuged at 13,000 × g for 5 min in an Eppendorf microcentrifuge at 4°C. An enzyme extract consisting of 100 mM Bicine pH 8.0, 25 mM KHCO_3_, 20 mM MgCl_2_, 3.5 mM ATP, 0.25 mM NADH, 5 mM phosphocreatine (PC), 17.5 Units per ml creatine phosphokinase (CPK), and 5 Units per ml both of Glyceraldehyde-3-phosphate-dehydrogenase (G3PD) and 3-phosphoglyceric phosphokinase (PGP) was immediately added to the reaction mixture to a final volume of 3 ml and incubated for 15 min at room temperature. The Rubisco activity was then measured at 340 nm using a spectrophotometer (UV-2550, Shimadzu, Kyoto, Japan). The reaction was initiated by addition of 0.25 mM RuBP (Ribulose bisphosphate).

### DCMU Application

To test the effects of reduced fruit photosynthesis in plants, the fruits were treated with the photosynthesis inhibitor DCMU from 65 DAP to maturation in 2016 (DCMU = 3-(3,4-dichlorophenyl)-1, 1-dimethy-lurea). DCMU is a chemical that is commonly used to inhibit Photosystem II of photosynthesis as it binds to the D1 protein in the reaction center (Allen and Holmes, 1986; Chow et al., 1990). The DCMU application has no other effects beyond the inhibition of photosynthesis (Fan et al., 2007).

The DCMU treatments were carried out by covering of the fruits with soaked paper immersed in 30 μM DCMU. The details of the treatments were determined on the basis of a preliminary experiment, where reduction of the functional PSII content (such as ETR) to ~50% treated by the soaked paper solution was found to occur after 30 min (**Table S1**). In the preliminary experiment, inhibition effect on ETR caused by DCMU application was assessed by a pulse-modulated fluorometer PAM 2500 (Walz, Effeltrich, Germany) 1 day after application. In detail, we made the ETR measurements in the middle part of the fruits where the surface is flat after collecting them from the trees. The distance between the fiber probe and the fruit surface was carefully kept constant in all measurements. In the preliminary experiment, the DCMU application significantly decreased the fresh weight and dry weight of ph fruits (**Table S2**).

In the main experiment, approximately 80 labeled ph and pp fruits from each of the six sampled trees were divided into two groups for the measurement. One group was treated with DCMU, and the other group was used as a control. In detail, 30 μM DCMU was dissolved in ethanol, and then mixed with 0.1% (v/v) Tween-20 as a surfactant. A tissue paper was first immersed in the DCMU solution for 2 min until it was entirely moist, then it was held it the air to allow excess solution to drain off. The experimental fruits were treated with the 30 μM of DCMU-soaked paper for 30 min. Control fruits were treated with paper soaked with Tween-20 alone solution at the same concentration. During the treatment, the leaves were not allowed to be in contact with the DCMU solution. The DCMU treatment was applied at midday on sunny days (any rain may dilute the solution) three times a week from 65 DAP until the fruits had reached maturity.

### Dry Weight and Shape Measurements of the Fruits After DCMU Application

On August 31st in 2016, the fruits were harvested, and the length, width, and the fresh weight (FW) of the intact fruits were measured immediately. Then the peels were removed from the fruits, and the fresh weight of the remaining hard fruit was measured. After drying to a constant weight, the dry weights (DW) of both the pseudo-peel and the hard fruit were measured. The sum of these two was taken as the dry weight of the intact fruits. The relative contribution of the fruit photosynthesis to the dry weight of the fruits was calculated as the percentage of the dry weight of the DCMU treated fruits, out of that of the control fruits. This was carried out separately both to the intact fruits and to the fruits with the peel removed.

### Oil and Sugar Content Measurements of the Kernel After DCMU Application

In order to measure the oil content and the soluble sugar content, the kernels of the fruits were ground with liquid nitrogen in a pestle and mortar. Approximately 8 g (per extraction thimble made of thick filter paper) of full fat flour was defatted in a Soxhlet apparatus with petroleum ether solvent (boiling point range 38.7°C–54.8°C) for 8 h. The defatted samples were dried overnight (10−12 h) in a fume hood to remove residual petroleum ether and then weighed to calculate the oil content. The soluble sugar content was assayed using the method of Zheng (2006) with modification. Dry flour samples (1 g) were ground with 20 ml of 80% (v/v) alcohol for 30 min. Following centrifugation at 10,000 × g for 15 min, the supernatant was transferred into a 100-ml volumetric flask. Then 20 ml of distilled water was added to the residue, which was centrifuged at 10,000 × g for 15 min, and then the supernatants were combined into a 100-ml volumetric flask to make 100 ml of solution for assays. Then, 0.5 ml of the assay solution was mixed with 0.5 ml of distilled water and 5 ml of anthrone reagent in turn into the tubes, which were then placed in a boiling water bath for 10 min. After cooling, samples were kept in the dark for 10 min to test the absorbance at the wavelength of 620 nm.

### Data Analysis

Data were subjected to analysis of variance (ANOVA) using SPSS statistical software (version 16.0, IBM, New York, USA). The data are presented as the mean ± SE. Differences at P ≤ 0.05 were considered significant.

## Results

### Appearances of the Fruits

**Figure 1:**
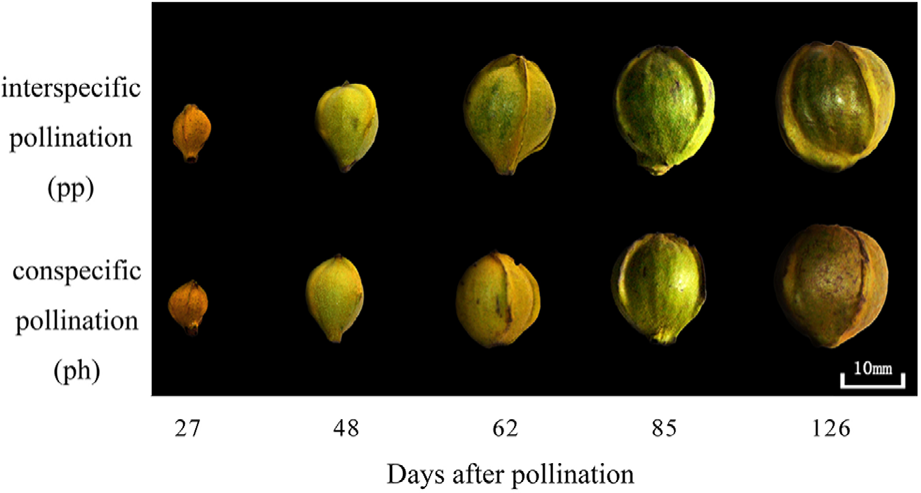
Differences at different fruit growth stages in 2016 in the appearance between hickory (*Carya cathayensis*) fruits developed either after intraspecific hickory pollination (ph fruits), or after interspecific pollination by pecan (pp fruits).

**Figure 2:**
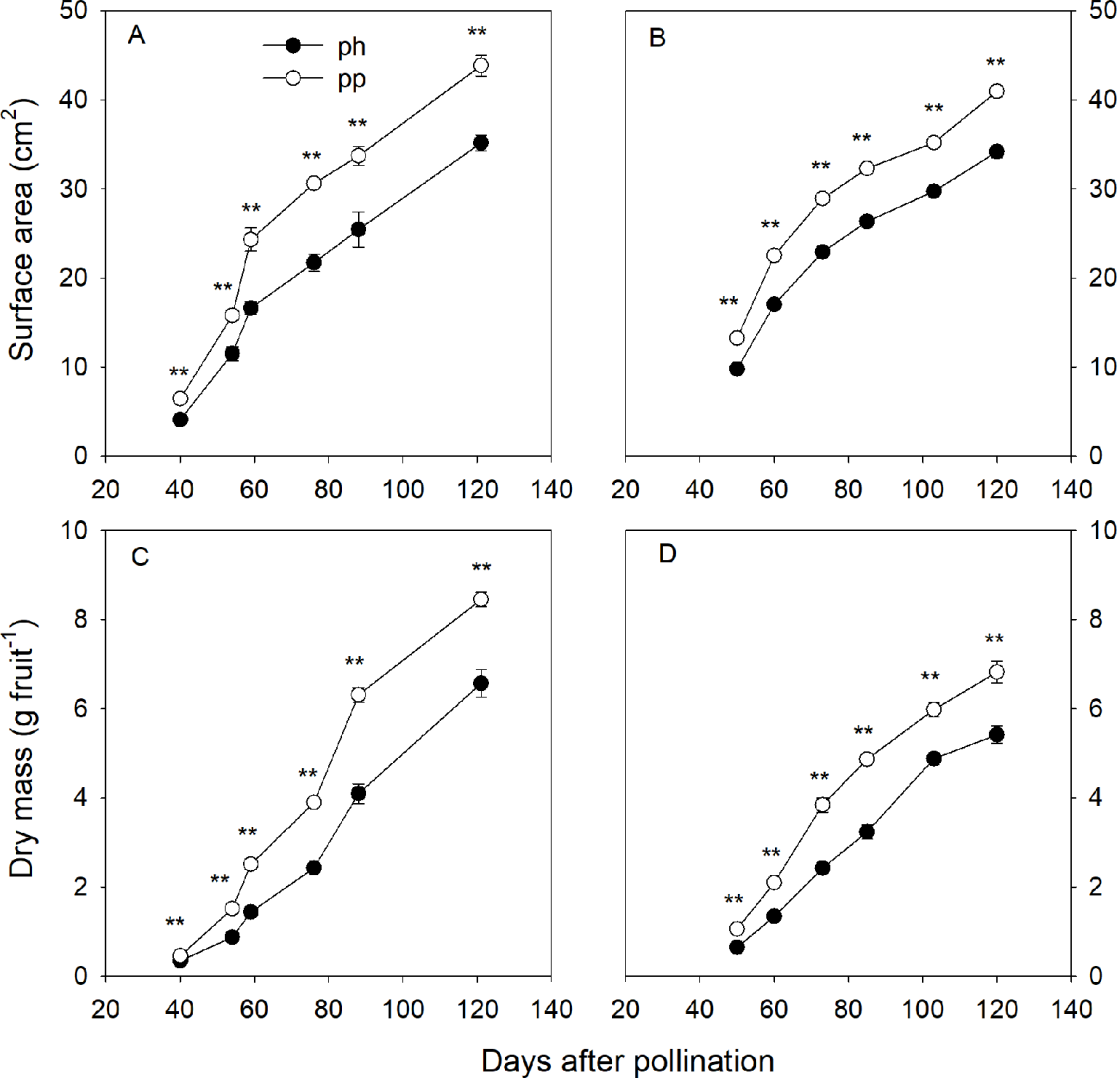
Surface area **(A, B)** and dry mass **(C, D)** of hickory (*Carya cathayensis*) fruits developed either after intraspecific hickory pollination (ph fruits), or after interspecific pollination by pecan (pp fruits), in 2015 **(A, C)** and 2016 **(B, D).** Significance of the differences between the ph and pp fruits: **, *P* < 0.01; n = 4 trees.

At 50 DAP the pp fruits were larger than the ph fruits, and the peel of the former was greener than that of the latter (**Figure 1**). Regardless of pollen parent, the surface area and dry weight gradually increased during the entire developmental time of the fruit (**Figure 2**). In 2015, the surface area of ph fruits and pp fruits increased by 304% and 276% from 40 to 59 DAP, respectively. In 2016, they increased correspondingly by 73.7% and 70.0% from 50 to 60 DAP. Similar trends were also found at the later growth stages. For instance, the surface area increased in 2015 from 59 to 121 DAP in ph and pp fruits by 112% and 80%, respectively, and in 2016 from 60 to 120 DAP correspondingly by 101% and 82%. The surface area and dry weight of pp fruits were higher than those of ph fruits. In 2015, the surface area of pp fruits was 4.3 and 7.7 cm^2^ larger than that of ph fruits at 54 and 59 DAP, respectively (**Figure 2A**). Similar differences in the surface areas between pp and ph fruits were found in 2016 at 50 DAP (3.4 cm^2^) and 60 DAP (5.5 cm^2^) (**Figure 2B**). In 2015, the dry weight of the ph fruits and the pp fruits increased from 59 to 121 DAP significantly by 5.7 g (equal to 78% of the final maturation dry weight) and 6.9 g (equal to 70% of the final maturation dry weight), respectively (**Figure 2B**). Similarly, in 2016, the dry weight of ph and pp fruits increased from 60 to 120 DAP by 4.0 g (equal to 75% of the final maturation dry weight) and 4.7 g (equal to 69% of the final maturation dry weight), respectively.

### The Gas Exchange in Fruits and Their Associated Leaves

**Figure 3:**
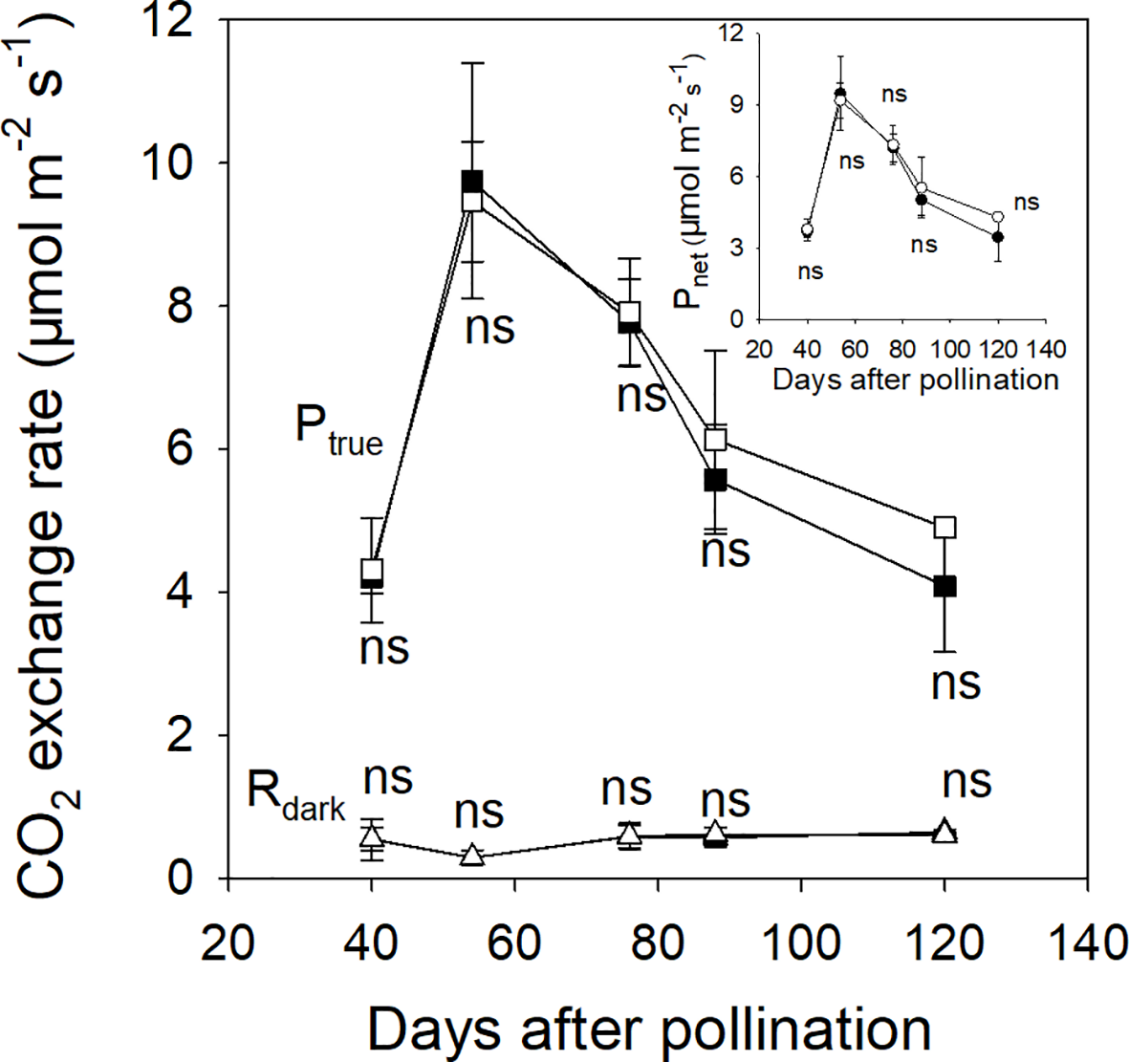
The true photosynthetic rate (P_true_, squares), the dark respiration rate (R_dark_, triangles) and the net photosynthetic rate (P_net_, circles in insert) of intraspecific hickory (*Carya cathayensis*) leaves associated to fruits developed either after intraspecific hickory pollination (ph fruits, closed symbols), or after interspecific pollination by pecan (pp fruits, open symbols), at the different fruit growth stages in 2015. Significance of the differences between the leaves associated to ph and pp fruits: ns, *P* > 0.05. n = 4 trees.

**Figure 4:**
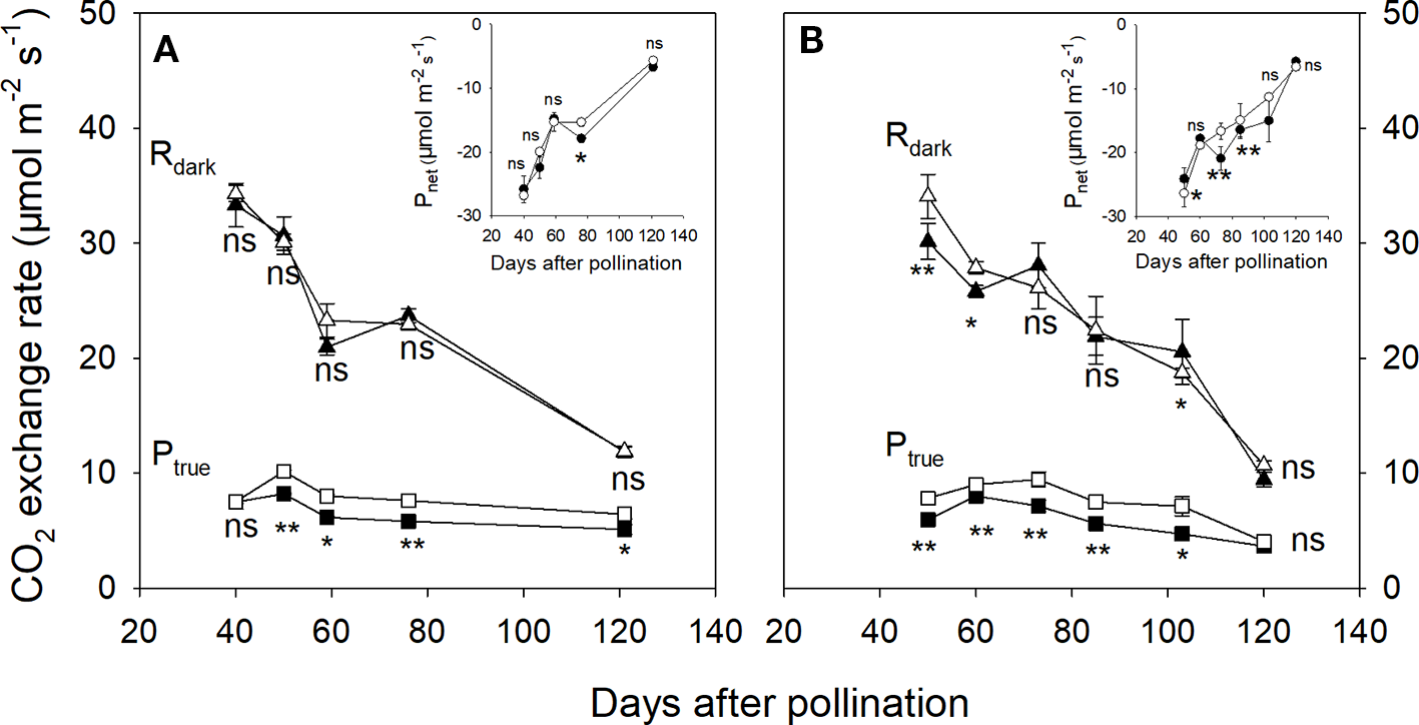
The true photosynthetic rate (P_true_, squares), the dark respiration rate (R_dark_, triangles), and the net photosynthetic rate (P_net_, circles in insert) of hickory (*Carya cathayensis*) fruits developed either after intraspecific hickory pollination (ph fruits, closed symbols), or after interspecific pollination by pecan (pp fruits, closed symbols), at different fruit growth stages in 2015 **(A)** and 2016 **(B)**. Significance of the differences between the ph and pp fruits: **, *P* < 0.01; *, *P* < 0.05; ns, *P* > 0.05. n = 4 trees.

In 2015, the associated leaves of ph and pp fruits reached peak values of P_true_ and P_net_ at about 54 DAP (**Figure 3**). After that, the P_true_ of the associated leaves significantly decreased by 58.1% until 120 DAP (**Figure 3**). There was little change in the R_dark_ of the associated leaves of both ph and pp fruits during fruit development. Both fruit types showed a significant decreasing trend of R_dark_, and an increasing trend of P_net_ (**Figure 4**). The R_dark_ of the ph and pp fruits decreased in 2015 from 40 to 59 DAP by 37.0% and 32.0%, and in 2016 from 50 to 60 DAP by 14.4% and 18.2%, respectively. Similar trends of R_dark_ were also found at the later growth stages. For instance, R_dark_ decreased in 2015 from 59 to 121 DAP in ph and pp fruits by 43.5% and 48.8%, respectively, and in 2016 correspondingly by 63.4% and 61.7% from 60 to 120 DAP. The P_true_ of both ph and pp fruits showed an initial increasing trend followed by a decreasing trend as the fruit development progressed (**Figure 4**). The P_true_ of pp fruits was 1.23–1.31 fold (*P* < 0.05) and 1.10–1.51 fold (*P* < 0.05) higher than that of ph fruits during the most development stages in 2015 and 2016, respectively.

### The Chloroplast Ultrastructure in the Peel of Fruits

**Figure 5:**
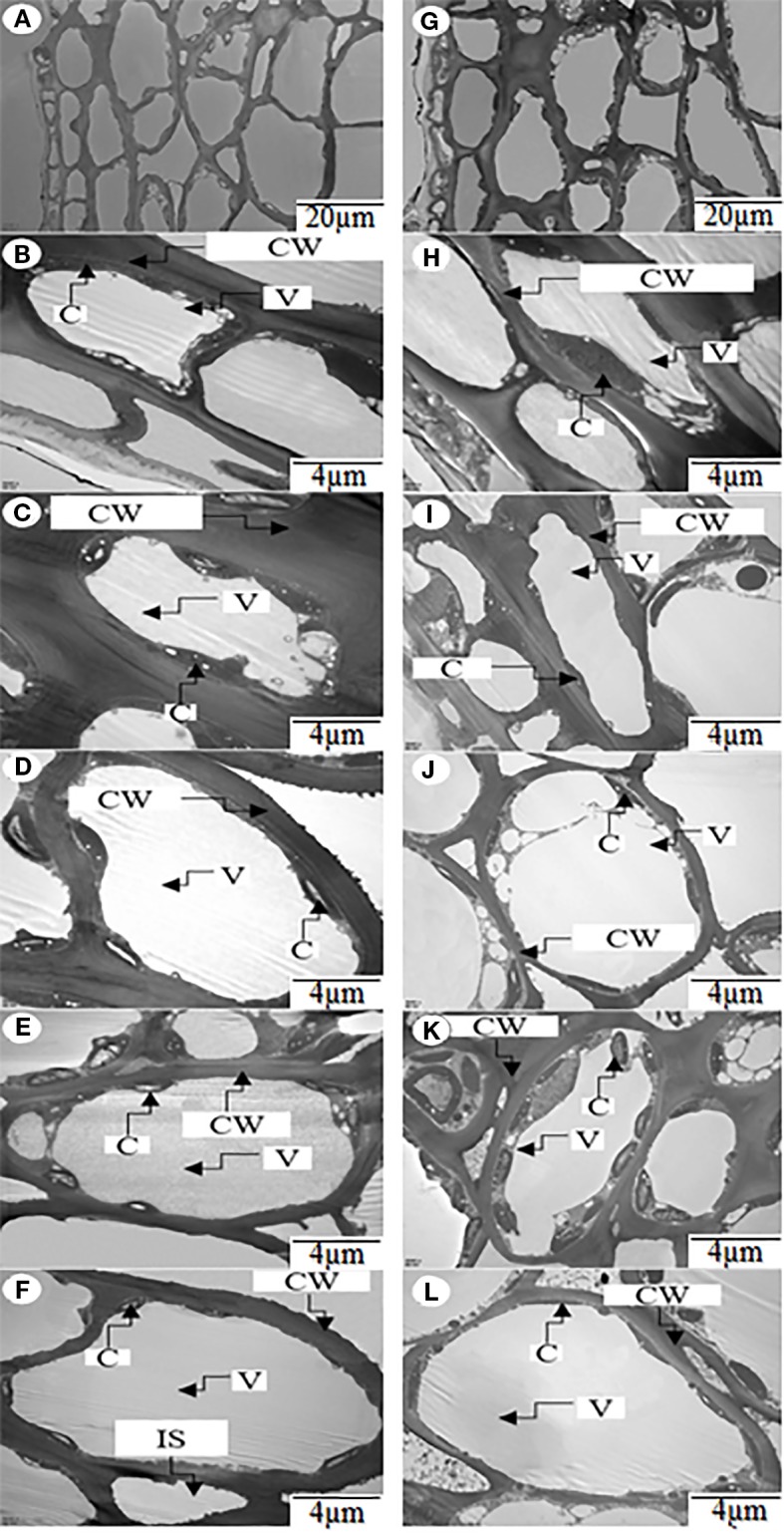
Ultrastructure of the chloroplasts in the zone from the first to the sixth cell layer of peels of hickory (Carya cathayensis) ph fruits (intraspecific pollination by hickory) and pp fruits (interspecific pollination by pecan) at 84 DAPS in 2017. **(A)**, an overview of the first six cell layers of peel in ph fruits; **(G)**, an overview of the first six cell layers of peel in pp fruits; **(B**– **F)**, the ultrastructures of the chloroplasts in each of the second to the sixth cell layers of peel in ph fruits; **(H**–**L)**, the ultrastructures of the chloroplasts in each of the the second to sixth cell layers of peel in pp fruits. CW, cell wall; C, chloroplast; V, vacuole.

We investigated the number of chloroplasts per cell in the uppermost six layers of peel of the ph and pp fruits (**Figure 5**). Both in ph and pp fruits, the cell size was significantly larger in the lower than in the upper layers (**Figure 5** and **Table 1**). The size of the first layer cells of pp fruits was significantly larger than that of ph fruits (*P* = 0.002). Surprisingly, in the other layers we found only few differences in the cell sizes between ph and pp fruits (except in the 5th layer). Both in ph and pp fruits, the chloroplast density per cell was significantly higher in the lower than in the upper layer cells (**Figure 5** and **Table 1**). In several layers (the 3^rd^, 5^th^, and 6^th^ cell layer), the number of chloroplasts per cell were 1.14-1.34 fold higher in the pp than in ph fruits (*P* < 0.05). Except in the 2^nd^ cell layer, the sizes of the chloroplasts in the other cell layers of peels were 1.25–1.83 fold greater in the pp than in the ph fruits (*P* < 0.05).

**Table 1:** Chloroplast characters at 84 DAP in 2017 in the zone from the first to the sixth cell layer in peels of hickory (*Carya cathayensis*) fruits developed either after intraspecific hickory pollination (ph fruits), or after interspecific pollination by pecan (pp fruits).

Cell layer	Size of cell (µm2)		Number of chloroplasts per cell		Chloroplast size (µm2)	
	ph fruits	pp fruits	ph fruits	pp fruits	ph fruits	pp fruits
**1^st^**	29.36 ± 5.72^b^	54.35 ± 13.22^a^	–	–	–	–
**2^nd^**	75.50 ± 5.98^a^	82.34 ± 11.81^a^	2.50 ± 0.50^a^	2.63 ± 0.50^a^	1.03 ± 0.09^a^	1.89 ± 0.62^b^
**3^rd^**	211.20 ± 37.24^a^	190.08 ± 27.56^a^	3.08 ± 0.25^b^	4.13 ± 0.13^a^	3.05 ± 0.69^a^	2.91 ± 0.38^a^
**4^th^**	382.64 ± 22.61^a^	405.05 ± 91.44^a^	4.63 ± 0.13^a^	4.65 ± 0.22^a^	3.11 ± 0.23^b^	3.89 ± 0.62^a^
**5^th^**	387.18 ± 14.57^b^	589.18 ± 37.85^a^	5.00 ± 0.50^b^	6.42 ± 0.42^a^	4.55 ± 0.81^b^	5.78 ± 0.37^a^
**6^th^**	489.45 ± 115.31^a^	525.53 ± 171.86^a^	6.65 ± 0.36^b^	7.59 ± 0.02^a^	3.49 ± 0.45^b^	4.44 ± 0.47^a^

Different letters indicate significant differences at P < 0.05 level. n = 4 trees.

### The Chlorophyll Content in the Peel

**Figure 6:**
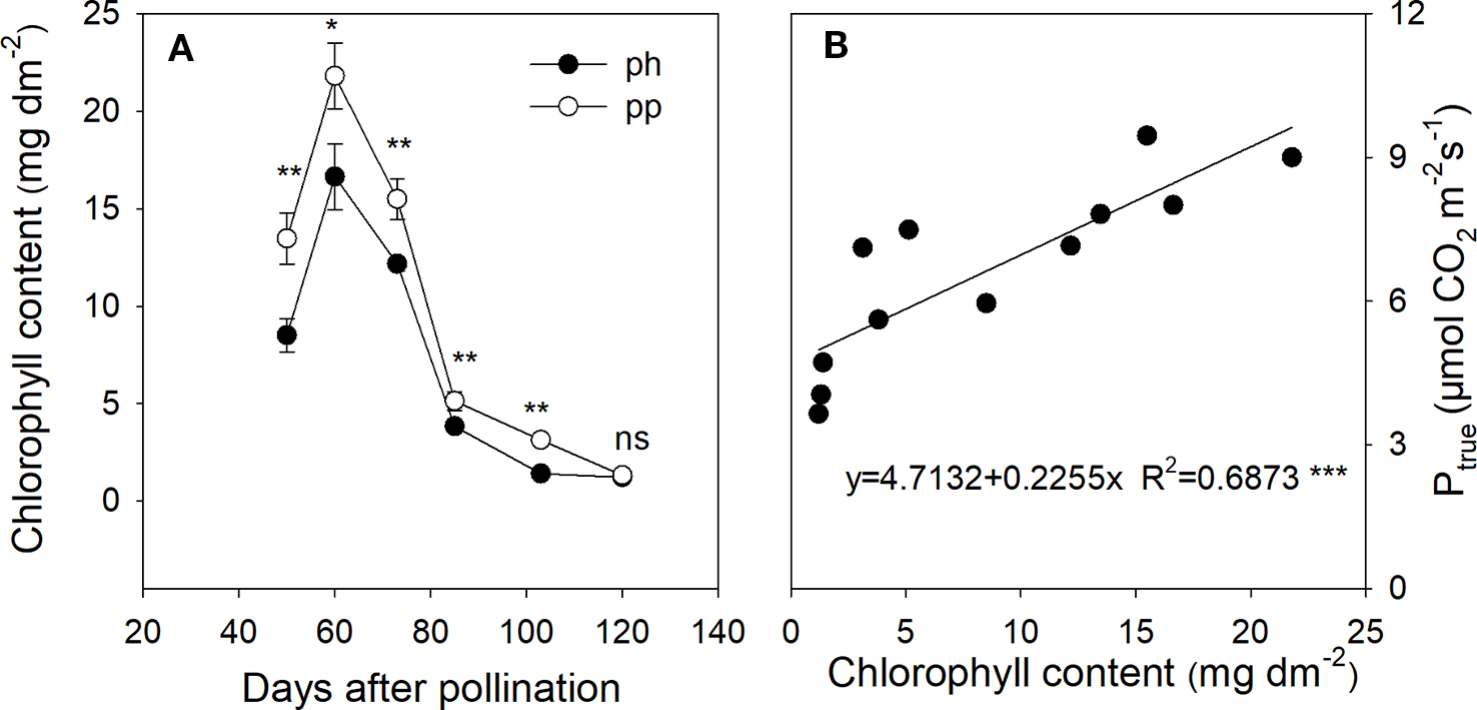
Chlorophyll content in peels of ph fruits (intraspecific pollination by hickory, closed symbols) and pp fruits (interspecific pollination by pecan, open symbols) of hickory (*Carya cathayensis*) at the different fruit growth stages in 2016 **(A)**, and correlation between the true photosynthetic rate and chlorophyll content during the same time period in both types of fruits **(B)**. Significance of the differences between ph and pp fruits:***, *P* < 0.001; **, *P* < 0.01; *, *P* < 0.05; ns, *P* > 0.05. n = 4 trees.

In both ph and pp fruits, the chlorophyll content of peels showed a significant initial increase followed by a rapid decrease with age (**Figure 6A**). Both fruit types reached a chlorophyll peak at 60 DAP, after which the chlorophyll content decreased by 77% from 60 to 85 DAP, and then decreased further by 68% from 86 to 120 DAP. From 50 to 103 DAP, the chlorophyll content was significantly higher in the pp than in the ph fruits (*P* < 0.05), but there was no significant difference between these two fruit types at 120 DAP. The chlorophyll content was significantly and positively correlated with P_true_ (**Figure 6B**).

### The Nitrogen Content in the Peel of Fruits

**Figure 7:**
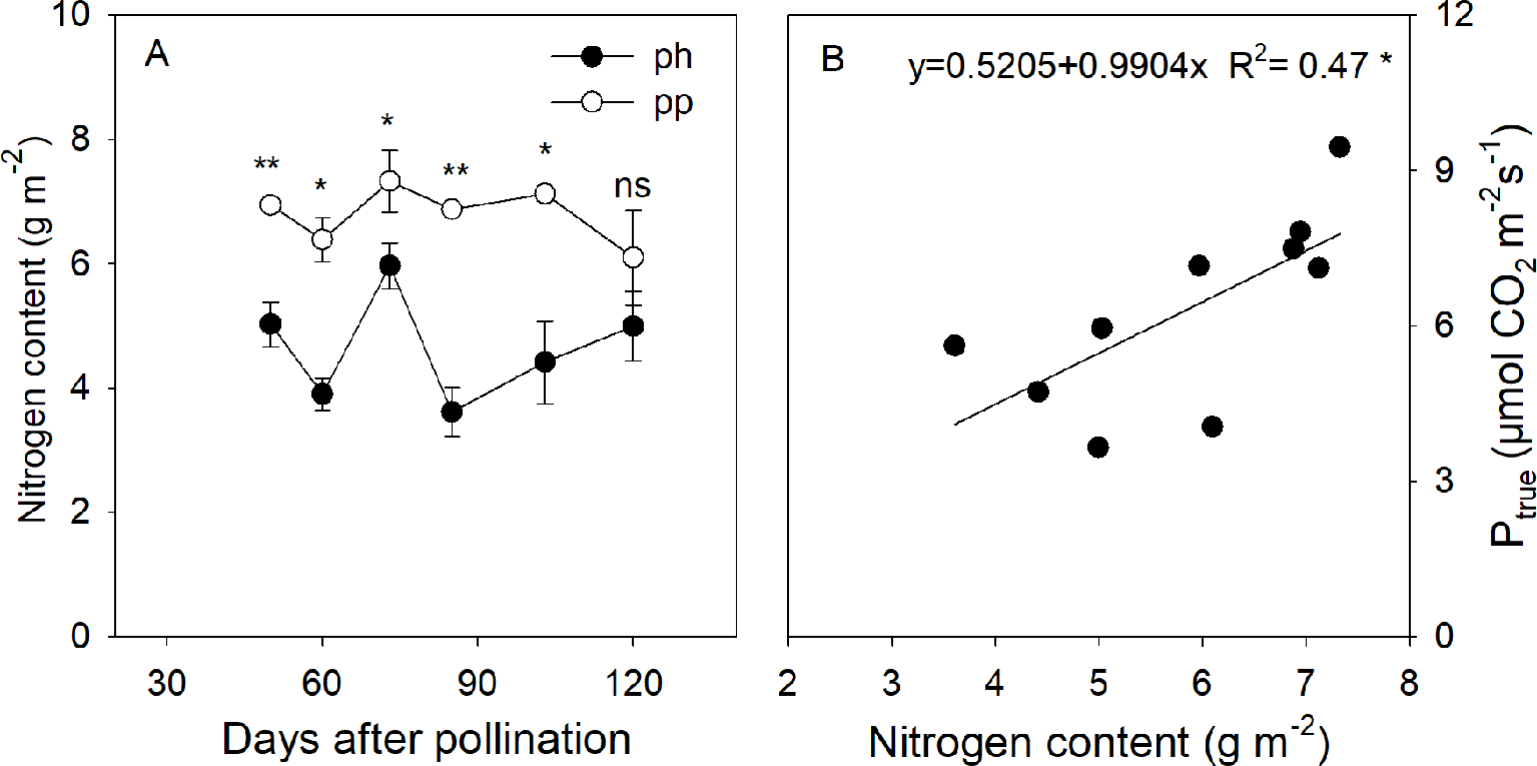
Nitrogen content in peels of ph fruits (intraspecific pollination by hickory, closed symbols) and pp fruits (interspecific pollination by pecan, open symbols) of hickory (*Carya cathayensis*) at the different fruit growth stages in 2016 **(A)**, and correlation between the true photosynthetic rate and nitrogen content during the same time period in both types of fruits **(B)**. Significance of the differences between the ph and pp fruits: **, *P* < 0.01; *, *P* < 0.05; ns, *P* > 0.05. n = 4 trees.

The nitrogen content of peels in the pp fruits was 1.27–2.23 fold (*P* < 0.05) higher than that in the ph fruits during the fruit development stages (**Figure 7A**). In the pp fruits, the nitrogen content significantly decreased from 103 to 120 DAP. The nitrogen content was linearly and positively correlated with P_true_ of fruits (**Figure 7B**).

### The Rubisco Activity in the Peel of Fruits

**Figure 8:**
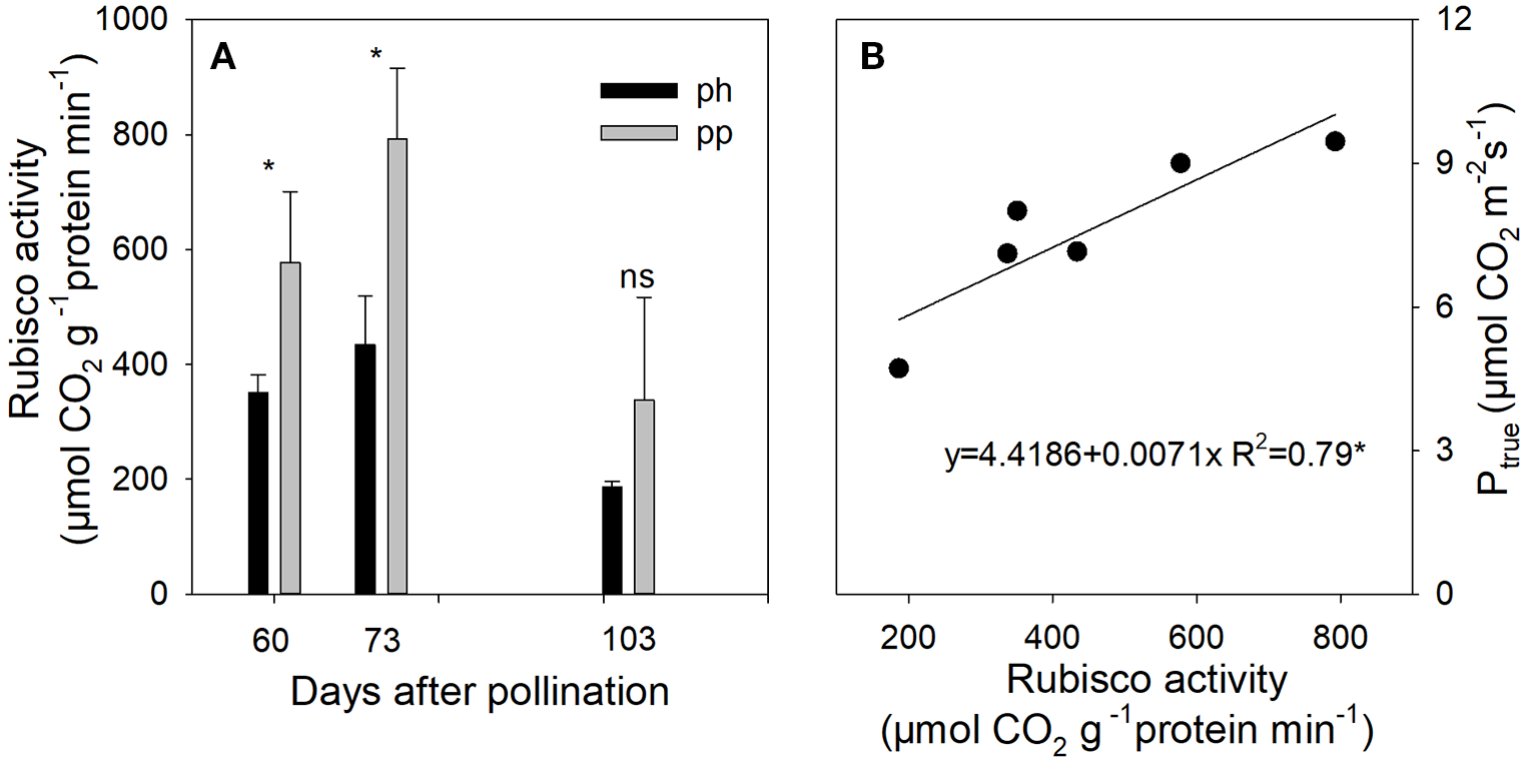
Rubisco activity in peels of ph fruits (intraspecific pollination by hickory, closed symbols) and pp fruits (interspecific pollination by pecan, open symbols) of hickory (*Carya cathayensis*) at the different fruit growth stages in 2016 **(A)**, and correlation between the true photosynthetic rate and Rubisco activity during the same time period in both types of fruits **(B)**. Significance of differences between the ph and pp fruits: *, *P* < 0.05; ns, *P* > 0.05. n = 4 trees.

From 60 to 73 DAP, the Rubisco activity in peel of pp fruits was 1.65–1.83 fold (*P* < 0.05) higher than that of ph fruits (**Figure 8A**). The Rubisco activity was significantly and positively correlated with P_true_ (**Figure 8B**).

### Effects of DCMU Incubation on the Dry Weight and Quality of Fruits

In the ph fruits, the relative contribution of fruit photosynthesis to the dry matter accumulation of the whole fruits and kernels (without peel) was 10% and 11%, respectively (**Table 2**). In the pp fruits, the corresponding percentages were 16.5% and 27.8%, respectively. The DCMU application significantly reduced the oil content of kernels by about 7.5% and 5.1% in the ph and pp fruits, respectively. Furthermore, the soluble sugar content of kernels decreased by 30% in both fruit types when treated with DCMU (**Table 2**).

**Table 2:** Effects of photosynthesis inhibitor (DCMU) on hickory (*Carya cathayensis*) fruits developed either after intraspecific hickory pollination (ph fruits), or after interspecific pollination by pecan (pp fruits). CK denotes control.

Parameter	ph fruits		pp fruits	
	CK	DCMU	CK	DCMU
**Fresh weight (g)**	18.7 ± 0.4^*^	17.10 ± 0.6	22.5 ± 0.3^*^	21.2 ± 0.5
**Dry weight (g)**	6.9 ± 0.2^*^	6.2 ± 0.2	8.5 ± 0.2***	7.1 ± 0.2
**Dry weight with peel removed (g)**	4.5 ± 0.1*	4.0 ± 0.2	5.4 ± 0.1***	3.9 ± 0.2
**Oil content (%)**	67.0 ± 0.1^*^	62.0 ± 2.8	68.8 ± 0.7**	65.3 ± 0.5
**Soluble sugar content (%)**	28.3 ± 0.6***	19.7 ± 0.5	31.3 ± 1.0***	22.2 ± 0.5

Significance of the differences between the ph and pp fruits: ***, P < 0.001; **, P < 0.01; *, P < 0.05; ns, P > 0.05. n = 6 trees.

## Discussion

### A Relatively Larger Difference in the Growth Between Ph and Pp Fruits At the Late Growth Stage

Earlier morphological studies of hickory fruits pollinated with interspecific pecan pollen have shown much larger size and greener color in the fruits developed after interspecific than in those developed after intraspecific pollination (Li et al., 1982; Wang et al., 2010). This is consistent with the observations on morphological traits in the present study (**Figure 1**). We observed a relatively larger difference in the surface area between ph and pp fruits after about 60 DAP than during the earlier growth stages (**Figures 2A, B**). According to the growth pattern of fruit dry weight, 70%−78% of dry matter accumulation was completed during the later growth stages (after about 60 DAP, **Figures 2C, D**). Consequently, studying the factors responsible for the difference in dry weight accumulation during the later growth stage is necessary for the understanding of the mechanisms of metaxenia for the enlarged fruits of *C. cathayensis*.

### The Enlargement of Pp Fruits May Due to the Enhanced Photosynthetic Capacity of the Peels

Amplified fragment length polymorphism (AFLP) and simple sequence repeat (SSR) analyses have found no obvious differences in the phenotype between offspring seedlings and the mother tree (Wang et al., 2010). Moreover, the embryos originated from nucellar cells of the maternal plant (mother plant) and were thus not the result of the fertilization of female cells by male cells (Zhang et al., 2012). These results indicated that the interspecific offspring seedlings were not true hybrids and that the variation in fruit characteristics was not caused by genetic changes. Fruits are sink tissues that need to import carbohydrates from photosynthetically active source tissues (Blanke and Lenz, 1989; Gallardo et al., 2008; Cocaliadis et al., 2014). Although green leaves are generally considered as the main sources of photosynthate production, numerous sources of evidence suggest that reproductive organs can capture light energy to perform photosynthesis and that this is an important additional contribution to carbon acquisition and yield (Whiley et al., 1992; Aschan and Pfanz, 2003; Hu et al., 2012; Hua et al., 2012; Hu et al., 2018; Brazel and Ó'Maoiléidigh, 2019). In the present study, the dry weight of the fruit was the sink capacity, and the photosynthesis of leaves and fruits were the sources. There was no significant difference in P_true_ or P_net_ of the associated leaves during the fruit development stages between fruits pollinated by different pollen parents (**Figure 3**), indicating that pollen has some effect on an important photosynthetic source (the photosynthesis of the pericarp) directly associated with fruit development. A plant may alter its photosynthesis to re-balance the source-sink ratio when carbohydrate levels reach a threshold value in the leaves (Paul and Foyer, 2001). Accordingly, we suggest that larger size of pp fruits enhances their fruit own photosynthesis, and in this way facilitates the accumulation of more carbohydrates for their further fruit development.

In the present study, the leaf P_true_ per leaf area significantly decreased by 48%−58% during 54−120 DAP, suggesting that the photosynthesis of fruits plays an important role in fruit development during the late stages of fruit development (**Figure 3**). Indeed, we observed less reduction of the photosynthetic rate in fruits than in leaves during the late fruit development stage, and this was accompanied by significantly increased surface area of fruits in 2015 (**Figures 2** and **4**). This was consistent with the finding of Hu et al. (2012) who reported that the photosynthesis of non-foliar green organs in cotton plays an important role in yield formation at the late growth stage. Furthermore, in the present study pp fruits had a significantly higher P_net_ per area accompanied by significantly larger surface area at the later fruit development stages (**Figures 2** and **4**), indicating that the photosynthesis of fruits plays an important role in the contribution to the increased dry mass of the pp fruits.

Respiration provides the energy for the synthesis of new phytomass (Amthor, 2000). In the present study, the R_dark_ per area of the fruits significantly decreased during fruit development, and this was accompanied by an increased surface area of the fruits (**Figures 2** and **4**). These findings indicate that the larger increase in surface area of fruits compensates for their decreasing R_dark_, supporting the respiratory cost of synthesis of new biomass.

### The Metaxenia Effect on the Photosynthetic Characteristics During the Growth Stages

Chloroplasts are the essential organelles for plant photosynthesis, which is important for amino, lipid, and phytohormone production and for starch and oil storage (Sakamoto et al., 2008). Cocaliadis et al. (2014) suggested that engineering fruit chloroplasts by transcriptional tools in tomatoes could improve fruit quality during the development and ripening stages. In the present study, the significantly increased numbers of chloroplasts per cell in the lower layers of the peel might be due to the larger cell size (**Figure 5** and **Table 1**). The chloroplast number per cell is strongly correlated with the cell size (Pyke and Leech, 1992). In some cell layers, the size of cells, the chloroplast number per cell, as well as the size of the chloroplasts in the pp fruits were all significantly greater than those of ph fruits, suggesting that higher nitrogen content in peels of pp fruits might enlarge chloroplasts. High nitrogen supply has been shown to enlarge the chloroplast in mesophyll cells, which is very likely to prevent the rapid movement of chloroplasts (Xiong et al., 2015a). To test this hypothesis, further studies are necessary to explore the effects of nitrogen supply on the chloroplast development.

Chlorophyll plays an important role in the light absorption and energy transduction in photosynthesis. In our study, the trend of chlorophyll content in fruits pollinated with two different pollen types was mostly the same as the trend for P_true_ during the growth stages (**Figure 6A**). We also observed a significant positive correlation between the chlorophyll content and photosynthetic rate of fruits (**Figure 6B**), indicating that the increased photosynthetic rate of pp fruits might be due to their enhanced chlorophyll biosynthesis.

Nitrogen plays an important role in plant photosynthesis. In the present study, we found that the nitrogen content in peels was enhanced by interspecific pollination (**Figure 7A**), and that the gross photosynthetic rate was positive correlated with the nitrogen content (**Figure 7B**). A positive relationship between leaf nitrogen content per leaf area and photosynthetic rate has been reported in numerous previous studies (Yamori et al., 2011; Xiong et al., 2015b). Usually, more than half of the leaf nitrogen is allocated to photosynthetic proteins such as Rubisco (Evans, 1989). Numerous studies have shown that the amount or the activity of Rubisco is correlated with the photosynthetic rate (Evans, 1989; Reich and Walters, 1994; Priwitzer et al., 1998; Hrstka et al., 2005). Hence, Makino (2003) suggested that nitrogen content can be used as a qualitative measure of the Rubisco content. Our results showed that the nitrogen content and Rubisco activity in pp fruits were significantly higher than those in ph fruits, and this was accompanied by significant positive correlations between the nitrogen content, Rubsico activity, and photosynthetic rate (**Figures 7** and **8**). Ten differentially expressed proteins in the pericarp identified by MALDI-TOF-TOF/MS (matrix assist laser desorption/ionization—time—of flight-time of—flight/Mass spectrometry) were found between differently pollinated Duweiwendan pomelo trees, and the effects of pollen type on nitrogen assimilation, photosynthesis, and antioxidant activity could lead to abnormal fruit development (Li, 2009). Interestingly, the nitrogen content in pp fruits rapidly decreased during 103−120 DAP, whereas no significant change was observed in ph fruits. It has been reported that nitrogen in the foliage and pericarp of almonds is rapidly imported into the developing embryo (Weinbaum and Muraoka, 1986), and a similar phenomenon has been observed in pecans (Thor and Smith, 1935). Similarly, the faster decline in nitrogen content in the peel of pp fruits in the present study suggests that the enlargement of the fruits might be related to their greater amount of nitrogen-containing compounds being rapidly imported into the developing fruits during the later growth stage.

Inhibition of the fruit photosynthesis induced by DCMU application had a significant effect on the dry weight of whole fruits and kernels. Moreover, a relatively larger reduction was observed in the pp than in the ph fruits (**Table 2**), indicating that a greater source amount of assimilates in pp fruits is supplied by the fruit photosynthesis. Numerous studies have shown that the photosynthesis of reproductive organs can contribute to oil formation of seeds (Singal et al., 1995; Ruuska et al., 2004; Goffman et al., 2005; Allen et al., 2009; Hua et al., 2012; Wang et al., 2016; Hu et al., 2018). Indeed, we also observed that a significant reduction of oil content was induced by DCMU treatment due to a deficiency of the photosynthate supply from fruit photosynthesis.

In conclusion, our results showed that the enlarged pp fruit with increased dry weight was closely related to the enhanced photosynthetic capacity of those fruits. A relatively larger difference in surface area and dry weight was observed between ph and pp fruits at the later growth stage. Considering that the rapid decrease in P_true_ investigated in the corresponding leaves was not associated with any significant increase in their surface area, the relative contribution of leaves to the fruit growth appears to be decreased at the later growth stage. Moreover, the fruits showed less decrease of P_true_ per area, and that was accompanied with the increased surface area. Thus, the relative photosynthetic contribution of the fruits to the development of fruits was likely increased at the late growth stages. Our results showed that the number of chloroplasts per cell in several cell layers of fruits was significantly higher in pp fruits than in ph fruits. It appears that the upregulation of chlorophyll content, Rubisco activity, and nitrogen content is better coordinated in the pp than in the ph fruits, and that increases the dry matter accumulation of fruits at the late growth stage in the pp fruits. In addition, the inhibition of fruit photosynthesis *via* DCMU treatment resulted in a much larger reduction of dry weight of the whole fruit and kernels in pp than in ph fruits. These results suggest that the significantly increased dry matter accumulation of *C. cathayensis* fruits pollinated with interspecific *C. illinoinensis* pollen could be attributed to the enhanced photosynthetic capacity of the fruits.

## Data Availability Statement

All datasets generated for this study are included in the article/**Supplementary Material**.

## Author Contributions

Designing the work: YuH. Running the experiments: RH and YZ. Data analysis and statistics: RH and YZ. Article writing and revising: RH, YZ, QZ, JH, HH, YoH, YuH.

## Conflict of Interest

The authors declare that the research was conducted in the absence of any commercial or financial relationships that could be construed as a potential conflict of interest.
