# Supplementary material for: Photosynthetic Mechanisms of Metaxenia Responsible for Enlargement of Carya cathayensis Fruits at Late Growth Stages
Source: Front Plant Sci. 2020 Feb 19;11:84. doi: 10.3389/fpls.2020.00084 (PMC7058182; doi:10.3389/fpls.2020.00084)
Supplement: Supplementary file 1 [file DataSheet_1.docx]

Table S1 Effects of different concentrations of photosynthesis inhibitor DCMU on the ETR of *Carya cathayensis* fruits in comparison with the control (CK).

| **Treatment** | **Treatment length(min)** | ***ETR*（%**） |
| --- | --- | --- |
| **CK** | 15min | 100% |
|  | 30min | 100% |
| **DCMU1** | 15min | 100%±3.3 |
|  | 30min | 93.3±5.3 |
| **DCMU2** | 15min | 84.2%±2 |
|  | 30min | 48.3%±3.4 |

Note: DCMU1: 20 μM; DCMU2: 30 μM. Data indicate mean±SD. n=3 trees, 10 fruits from each tree.

Table S2 Effects of photosynthesis inhibitor (DCMU) on *Carya cathayensis* fruits developed after conspecific hickory pollination. CK = control.

| **Parameter** |  | |
| --- | --- | --- |
|  | **CK** | **DCMU** |
| **Fruit fresh weight**  **(g)** | 18.6±1.9^a^ | 15.2±1.8^b^ |
| **Fruit dry weights**  **(g)** | 5.9±0.6^a^ | 4.8±0.8^b^ |

Note: Different letters indicate significant differences at *P*<0.05 level. n=3 trees. 40 fruits from each tree.
